# Anatomic location of colorectal cancer presents a new paradigm for its prognosis in African American patients

**DOI:** 10.1371/journal.pone.0271629

**Published:** 2022-07-29

**Authors:** Donghai Wang, Raag Agrawal, Shuli Zou, M. A. Haseeb, Raavi Gupta

**Affiliations:** 1 Department of Pathology, State University of New York, Downstate Health Sciences University, Brooklyn, New York, United States of America; 2 Department of Pathology, Kings County Hospital Center, Brooklyn, New York, United States of America; 3 Department of Cell Biology, State University of New York, Downstate Health Sciences University, Brooklyn, New York, United States of America; OUHSC: The University of Oklahoma Health Sciences Center, UNITED STATES

## Abstract

Among all racial groups in the U.S., African Americans (AA) have the highest incidence of and mortality from colorectal cancer (CRC). Although socioeconomic factors, as the major contributors to racial disparity of CRC, have been widely investigated, there is a dearth of information germane to understanding its biological basis. To better elucidate the clinicopathologic features we extracted demographic, clinical, pathologic and molecular features of 500 consecutive cases of CRC diagnosed at our institution which has an AA-predominant patient population (75% of all patients). We compared data from our AA patients with those of white patients both from our institution and from SEER and the published literature for meaningful comparison. AA patients were more likely to be at an advanced disease stage (25.9% vs. 20.8%, p = 0.041), have low grade tumors (89.2% vs. 77.5%, p<0.001) in cecum (18.7% vs. 16.2%, p<0.001) and <60-years-old than white patients (31.8% vs. 26.3%, p = 0.015). The frequency of KRAS mutation was higher in AA patients than in white patients (56.8% vs. 20.7%, p<0.001). Amongst subtypes of KRAS tested in CRC, codon 12 mutation is more common in AA than white patients (85.2% vs. 68.9%, p = 0.020). Compared with other racial groups, we found AA patients to have worse disease-free survival (HR = 3.682, p = 0.035). Also, AA patients with CRC in distal (sigmoid and rectum) or proximal (cecum) colon have worse overall survival than those with CRC in middle colon (HR = 2.926, p = 0.014), a finding not observed in white patients. In both racial groups, advanced stage, perforation, and hypertension were independent prognostic factors for overall survival (p<0.05). Similarly, low body-mass index at presentation, mucinous adenocarcinoma, lymphovascular invasion, perineural invasion and KRAS mutations were independent factors significantly associated with poor disease-free survival. Collectively, our data provide new insights into the roles of clinicopathologic features, especially anatomic distribution, in predicting outcomes of CRC in AA population.

## Introduction

Colorectal cancer (CRC) is the third most common cancer and the second leading cause of cancer-related death in the United States. Racial disparities have long been recognized in this disease, given that African Americans (AA) have the highest incidence and mortality among all ethnic groups [1]. Large scale population-based studies credit improved prevention and treatment strategies, implemented since the early 2000s, with substantial declines in incidence and mortality of CRC [2, 3]. This trend has considerably reduced the difference in incidence of CRC between AA and white patients [2]. However, there is a persistent gap in survival of patients of the two racial groups, with the five-year survival among AA diagnosed between 2004 and 2009 not reaching the level observed among white patients diagnosed between 1990 and 1994, some 15 to 20 years earlier [3].

It is widely accepted that socioeconomic factors determine the access to screening programs and appropriate treatment for patients of different races, which in turn affects the stage of CRC at presentation and hence prognosis [4–6]. Nonetheless, the roles of other factors including tumor biology in predicting shorter survival among AA largely remains unknown. It is likely that distinct genetic or epigenetic mechanisms constitute the biological basis and have a combined effect on poor prognosis of CRC among AA patients [7–9]. We used a wide range of variables from a cohort of 500 patients diagnosed with CRC at our institution, and retrospectively analyzed the association of those features with race and factors that determine clinical outcomes. We also compared our data from AA patients with those from matched white patients using external data sources. Our study aims to identify biological or pathological predictors for prognosis of CRC among AA patients and underlying factors related to racial disparity in mortality from CRC.

## Methods

### Patient population and data source

We searched electronic medical records at our institution from 01/2009 to 12/2017 with the keyword “colorectal carcinoma”, “colorectal cancer”, “colon cancer”, or “colonic adenocarcinoma”. Tumors from anal canal, appendix, or with a histological type other than adenocarcinoma were excluded from the study. A total of 500 consecutive cases with histopathologic diagnosis of adenocarcinoma were retrieved. For survival analysis, we excluded data from patients with a history of other malignancies. Cancers arising from the rectum and rectosigmoid junction require neoadjuvant chemoradiotherapy before surgery and therefore are assigned “y” in Tumor Node and Metastasis (TNM) staging. These tumors were excluded from correlation analysis between races and stages, in which we only used“p” TNM staging group.

The study was approved by the Institutional Review Board and Privacy Board (IRB) of the State University of New York Downstate Medical Center [IRB: 546015].

Because of small number of white patients in our AA-predominant patient population, data of white patients were used from Surveillance, Epidemiology, and End Results (SEER) database to compare with our findings (https://seer.cancer.gov/statistics/; ver. 8.3.5). There are two types of approved SEER databases: public and specialty. We used the former for demographic, clinical and pathologic variables, while the latter was used for KRAS mutation status. The following algorithm was used for searching SEER: year of diagnosis: “2009–2015”; site: “colon and rectum”; diagnostic confirmation: “positive histology”; type of reporting source: not “autopsy only” or “death certificate only”; state: “Connecticut” or “New Jersey”. We also compared our KRAS and mismatch repair (MMR) data of AA patients with those of white patients from published studies [10–14], since neither data of KRAS subtypes nor data of MMR status are in major public databases.

### Study variables

Major demographic, clinical, pathologic, and molecular variables for the patient cohort were recorded. The demographic variables included age, sex, race; the clinical variables included: intestinal perforation, rectal bleeding, anemia, body weight, carcinoembryonic antigen (CEA) level at presentation, comorbidity (diabetes, hypertension, other cancers), adjuvant/neoadjuvant chemotherapy, adjuvant/neoadjuvant radiation, surgery; the pathologic variables included tumor location, size, histologic type, grade, stage, lymphovascular invasion (LVI), perineural invasion (PNI), intratumoral lymphocytic infiltration (ITL) and peritumoral lymphocytic infiltration (PTL); and molecular features included KRAS mutation and MMR status.

### Molecular testing

For KRAS mutation assay, genomic DNA was extracted from formalin-fixed, paraffin-embedded tumor sections using QIAmp Kit (QIAGEN, Valencia, CA) and amplified by polymerase chain reaction. DNA pyrosequencing was performed using PSQ HS 96 Gold SNP Reagents (Biotage, Uppsala, Sweden) with a PSQ HS 96A Pyrosequencer. Separate assays for detection of codons 12/13 and codon 61 were performed, using primers from the PyroMark KRAS kit (QIAGEN, Valencia, CA).

Expression of MMR proteins (MLH1, PMS2, MSH2, and MSH6) was analyzed in formalin-fixed, paraffin-embedded tumor sections using immunohistochemistry. Monoclonal antibodies included anti-MLH1 (clone G168-728), anti-PMS2 (clone MRQ-28), anti-MSH2 (clone G219-1129), and anti-MSH6 (clone 44). MMR protein loss was defined as the absence of nuclear staining in tumor cells in the presence of positive nuclear staining in normal colonic epithelium and stromal cells. Tumors were defined as MMR-deficient (dMMR) if one or more MMR proteins was lost, and MMR-proficient (pMMR) if all MMR proteins were detected.

### Statistical analysis

Statistical analyses were performed using SPSS (v. 18.0). Correlation analyses between clinicopathologic variables of different races were conducted by Chi-Square test. For survival analysis and to examine the impact of various factors on survival, overall survival (OS) and disease-free survival (DFS) were evaluated as outcome measurements. OS was defined as the time between initial diagnosis and death, and DFS as the time between curative surgery to first recurrence or death. Kaplan-Meier analysis was performed to compare survival differences relative to all variables. Variables with a p value of <0.2 were considered trending and were included in multivariate Cox proportional hazards model, which calculated hazard ratios (HR) and 95% confidence intervals (95% CI) for independent prognostic factors. Forward Stepwise method was used for selection of variables in analysis. Differences between variables with a p <0.05 were considered statistically significant.

## Results

### Epidemiology and clinicopathologic features

A total of 500 consecutive patients with CRC were included in the analysis. Racial/ethnicity information was available for 452 patients, of which 387 (85.6%) were AA. Most common age group that presented with CRC was ≥70 years in both AA and white patients. There was a male predominance amongst white patients in our institution (68.1% vs. 49.6%, p = 0.017), however, there was no significant difference in gender distribution in SEER database (p = 0.75). AA patients had a significantly higher frequency of tumors arising in cecum (18.7% vs. 16.2%, p<0.001), with advanced stage (25.9% vs. 20.8%, p = 0.041), with low grade differentiation (89.2% vs. 77.5%, p<0.001) and age younger than 60 years at the time of diagnosis (31.8% vs. 26.3%, p = 0.015). AA patients also tended to have a higher rate of hypertension (64. 9% vs. 51.1%, p = 0.064) and diabetes (33.1% vs. 23.9%, p = 0.208) than in white patients (Table 1).

**Table 1:** Clinicopathologic features of colorectal carcinoma in patients of different races.

Variable	Patients at this Institution, *n* (%)				SEER, *n* (%)	p	
	AA^a^ (*n* = 387)	White^b^ (*n* = 47)	Other (*n* = 16)	Unknown (*n* = 50)	White^c^(*n* = 33268)	a vs. b	a vs. c
Gender							
Male	192 (49.6)	32 (68.1)	9 (56.3)	24 (48.0)	16921 (50.9)	0.017	0.75
Female	195 (50.4)	15 (31.9)	7 (43.8)	26 (52.0)	16347 (49.1)		
Age (years)							
<40	11 (2.8)	1 (2.1)	1 (6.3)	2 (4.0)	713 (2.1)	0.761	0.178
40–49	29 (7.5)	5 (10.6)	0 (0)	5 (10.0)	2267 (6.8)		
50–59	83 (21.5)	13 (27.7)	3 (18.8)	13 (26.0)	5778 (17.4)		
60–69	116 (30.0)	13 (27.7)	5 (31.3)	16 (32.0)	7175 (21.6)		
≥70	148 (38.2)	15 (31.9)	7 (43.8)	14 (28.0)	17335 (52.1)		
<60	123(31.8)	19 (40.4)			8758 (26.3)	0.233	0.015
≥60	264 (68.2)	28 (59.6)			24510 (73.7)		
Body-Mass Index (kg/m^2^)							
<18.5	19 (5.7)	2 (5.6)	0 (0)	3 (6.8)		1.00	
≥18.5	315 (94.3)	34 (94.4)	12 (100.0)	41 (93.2)			
Hypertension							
Yes	251 (64.9)	24 (51.1)	6 (37.5)	27 (54.0)		0.064	
No	136 (35.1)	23 (48.9)	10 (62.5)	23 (46.0)			
Diabetes							
Yes	128 (33.1)	11 (23.9)	2 (12.5)	14 (28.0)		0.208	
No	259 (66.9)	35 (76.1)	14 (87.5)	36 (72.0)			
Other Cancer							
Yes	56 (14.5)	5(10.6)	0 (0)	3 (6.0)		0.475	
No	331 (85.5)	42 (89.4)	20 (100.0)	47 (94.0)			
Anemia							
Yes	293 (80.3)	32 (72.7)	8 (72.7)	33 (71.7)		0.242	
No	72 (19.7)	12 (27.3)	3 (27.3)	13 (28.3)			
Rectal Bleeding							
Yes	103 (26.6)	13 (27.7)	2 (12.5)	20 (40.0)		0.879	
No	284 (73.4)	34 (72.3)	14 (87.5)	30 (60.0)			
Intestinal Obstruction							
Yes	58 (15.0)	4 (8.5)	2 (12.5)	4 (8.0)		0.231	
No	329 (85.0)	43 (91.5)	14 (87.5)	46 (92.0)			
Intestinal Perforation							
Yes	15 (3.9)	2 (4.3)	1 (6.3)	1 (2.0)		1.00	
No	372 (96.1)	45 (95.7)	15 (93.8)	49 (98.0)			
Carcinoembryonic Antigen (ng/mL)							
≥5	127 (50.2)	11 (50.0)	4 (44.4)	12 (36.4)	8049 (48.7)	0.986	0.646
<5	126 (49.8)	11 (50.0)	5 (55.6)	21 (63.6)	8464 (51.3)		
Site of Carcinoma							
Cecum	68 (18.7)	6 (12.8)	3 (18.8)	6 (12.8)	5212 (16.2)	0.137	<0.001
Ascending colon	67 (18.4)	6 (12.8)	2 (12.5)	4 (8.5)	5303 (16.5)		
Transverse colon	28 (7.7)	2 (4.3)	1 (6.3)	2 (4.3)	2302 (7.2)		
Descending colon	29 (8.0)	5 (10.6)	0 (0)	7 (14.9)	1508 (4.7)		
Hepatic flexure	5 (1.4)	4 (8.5)	0 (0)	1 (2.1)	1048 (3.3)		
Splenic flexure	5 (1.4)	0 (0)	0 (0)	3 (6.4)	690 (2.2)		
Sigmoid colon	92 (25.3)	15 (31.9)	3 (18.8)	13 (27.7)	5994 (18.7)		
Rectum	70 (19.2)	9 (19.2)	7 (43.8)	11 (23.4)	10085 (31.4)		
Left	196 (53.8)	29 (61.7)	10 (62.5)	34 (72.3)	18277 (56.9)	0.309	0.248
Right	168 (46.2)	18 (38.3)	6 (37.5)	13 (27.7)	13866 (43.1)		
Proximal/Distal	230 (63.2)	30 (63.8)	13 (81.3)	30 (63.8)	21291 (66.2)	0.931	0.221
Middle	134 (36.8)	17 (36.2)	3 (18.8)	17 (36.2)	10852 (33.8)		
Histology							
Mucinous ADC	27 (7.1)	4 (8.5)	0 (0)	1 (2.0)	2656 (8.5)	0.958	0.325
Non-mucinous ADC	353 (92.9)	43 (91.5)	16 (100.0)	48 (98.0)	28506 (91.5)		
Grade							
WD	40 (12.7)	6 (14.6)	1 (7.1)	10 (27.0)	3022 (10.5)	0.83	<0.001
MD	240 (76.4)	32 (78.1)	11 (78.6)	21 (56.8)	19189 (66.9)		
PD	33 (10.5)	3 (7.3)	2 (14.3)	6 (16.2)	5755 (20.1)		
UD	1 (0.3)	0 (0)	0 (0)	0 (0)	703 (2.5)		
Stage (AJCC)							
1	54 (20.5)	9 (26.5)	3 (33.3)	10 (34.5)	6439 (25.3)	0.267	0.041
2	52 (23.6)	11 (32.4)	0 (0)	7 (24.1)	7009 (27.5)		
3	79 (30.0)	5 (14.7)	2 (22.2)	4 (13.8)	6727 (26.4)		
4	68 (25.9)	9 (26.5)	4 (44.4)	8 (27.6)	5302 (20.8)		
Lymphovascular Invasion							
Yes	62 (24.0)	9 (28.1)	3 (37.5)	1 (4.8)		0.611	
No	196 (76.0)	23 (71.9)	5 (62.5)	20 (95.2)			
Perineural Invasion							
Yes	28 (11.4)	7 (22.6)	1 (12.5)	1 (5.0)	2560 (13.1)	0.138	0.424
No	218 (88.6)	24 (77.4)	7 (87.5)	19 (95.0)	16962 (86.9)		
Intratumoral Lymphocytic Infiltration							
Yes	65 (53.3)	9 (50.0)	2 (100.0)	2 (40.0)		0.795	
No	57 (46.7)	9 (50.0)	0 (0)	3 (60.0)			
Peritumoral Lymphocytic Infiltration							
Yes	67 (54.5)	8 (44.4)	2 (100.0)	1 (16.7)		0.426	
No	56 (45.5)	10 (55.6)	0 (0)	5 (83.3)			

ADC, adenocarcinoma; AJCC, American Joint Committee on Cancer; MD, moderately differentiated; PD, poorly differentiated; SEER, Surveillance, Epidemiology and End Results program; UD, undifferentiated; WD, well differentiated

### KRAS mutation and MMR status

**Fig 1:**
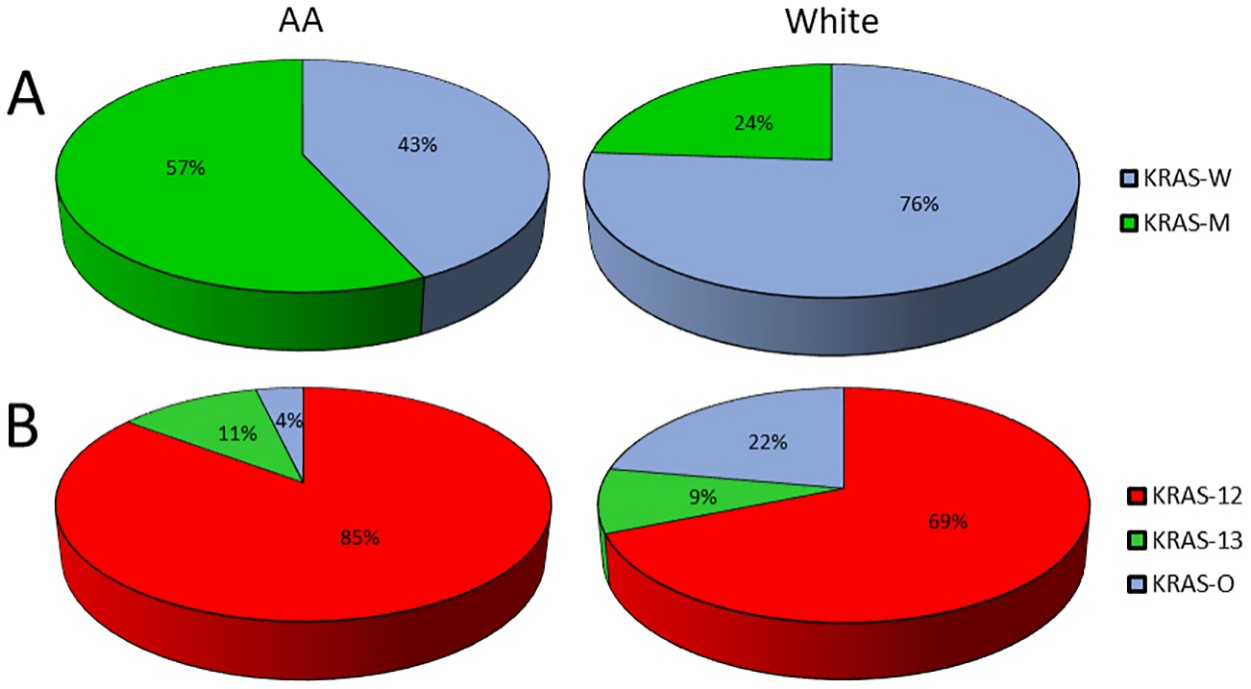
(A) Frequency of KRAS mutation is higher in AA compared with white patients. (B). Among all mutation tested, codon 12 mutation (KRAS-12) represents most common subtype for both AA and white patients, and has higher frequency in AA than white patients.

Overall frequency of KRAS mutation is increased in AA patients compared with those from the published literature and SEER data of white patients (p<0.001) (Table 2). Among all mutations tested, codon 12 mutation was more common in AA than in white patients (85.2% vs. 68.9%, p = 0.019) (Fig 1; Table 2). Analysis of conventional CRC locations (right and left) and our proposed CRC locations (proximal/distal and middle) showed that AA patients had significantly higher frequency of KRAS mutations than white patients both in left colon (51.9% vs. 10.7%, p<0.001) and in proximal/distal colon (55.9% vs. 26.4%, p<0.001) (S3 Table).

**Table 2:** Genetic profiles of patients and correlation with races.

Patients	Number of Patients (%) Expressing Tumor Markers									
	MSI	MSS	p^a^	KRAS-M	KRAS-W	p^b^	KRAS-12	KRAS-13	KRAS-O	p^c^
AA, our study	9 (15.5)	49 (84.5)		54 (56.8)	41 (43.2)		46 (85.2)	6 (11.1)	2 (3.7)	
White, our study	2 (25.0)	6 (75.0)	0.866	3 (50.0)	3 (50.0)	NA	3 (100.0)	0 (0)	0 (0)	NA
White, paper^10,13^	20 (8.8)	206 (91.2)	0.135	45 (23.9)	143 (76.1)	<0.001	31 (68.9)	4 (8.9)	10 (22.2)	0.019
White, paper^11,14^	39 (14.1)	237 (85.9)	0.784	42 (20.7)	161 (79.3)	<0.001				
White, paper^12^	21 (11.7)	159 (88.3)	0.442							
White, SEER				1117 (38.7)	1768 (61.3)	<0.001				

^a^. Comparison for MMR status

^b^. Comparison for KRAS mutation

^c^. Comparison for KRAS mutation subtypes

AA, African American; KRAS-M, mutant KRAS; KRAS-O, other subtypes of KRAS; KRAS-W, wild-type KRAS; MSI, microsatellite instability; MSS, microsatellite stability; NA, not available due to small sample size of white patients

For distribution of MMR status, there is no significant difference between AA and white patients, after comparing AA data with white patient data from published literatures (Table 2).

### Survival analysis for prognostic factors by race

We analyzed the association between all variables and survival (OS and DFS) with Kaplan-Meier analysis, by AA and all-race group, instead of individual races. This allowed examination and comparison of the effect of combined races and AA on the pattern of prognostic factors revealing the variables that distinctly affect prognosis in AA patients. Low BMI at presentation (<18.5 kg/m^2^), high CEA (≥5 ng/mL), intestinal perforation, advanced American Joint Committee on Cancer (AJCC) stage, and presence of LVI were significantly associated with shorter OS and higher frequency of event for both AA group and all-race group. The tumors from cecum, sigmoid colon and rectum appeared to relate to a shorter OS for all races. However, in AA patients, the trend became more prominent, although for both groups, the trend is non-significant. Intriguingly, when we used the term proximal colon (cecum), distal colon (sigmoid colon and rectum), and middle colon (ascending colon, hepatic flexure, transverse colon, splenic flexure, and descending colon), the analysis showed a more delineated separation of risk prediction for both AA group (p = 0.006) and all-race group (p = 0.064). Apparently, the OS of AA patients are more dependent on anatomic site of the tumor, than other races, with the proximal/distal location conferring a worse OS (S1 Table).

In AA population the incidence seems to increase steadily whereas in whites the incidence increases sharply after the 6^th^ decade of life. Another interesting finding of CRC of all races in different age groups was that patients in their 4^th^ decade had the best prognosis; those older showed a trend of decreasing OS with increasing age, so did patients younger than 40 (p = 0.037).

For DFS, analysis of both groups showed that low BMI, high CEA, advanced stage, KRAS mutation, LVI and PNI were significantly associated with poor prognosis. Unlike OS, the tumor site had no prognostic relevance for DFS (S2 Table).

### Analysis for independent prognostic factors by race

**Fig 2:**
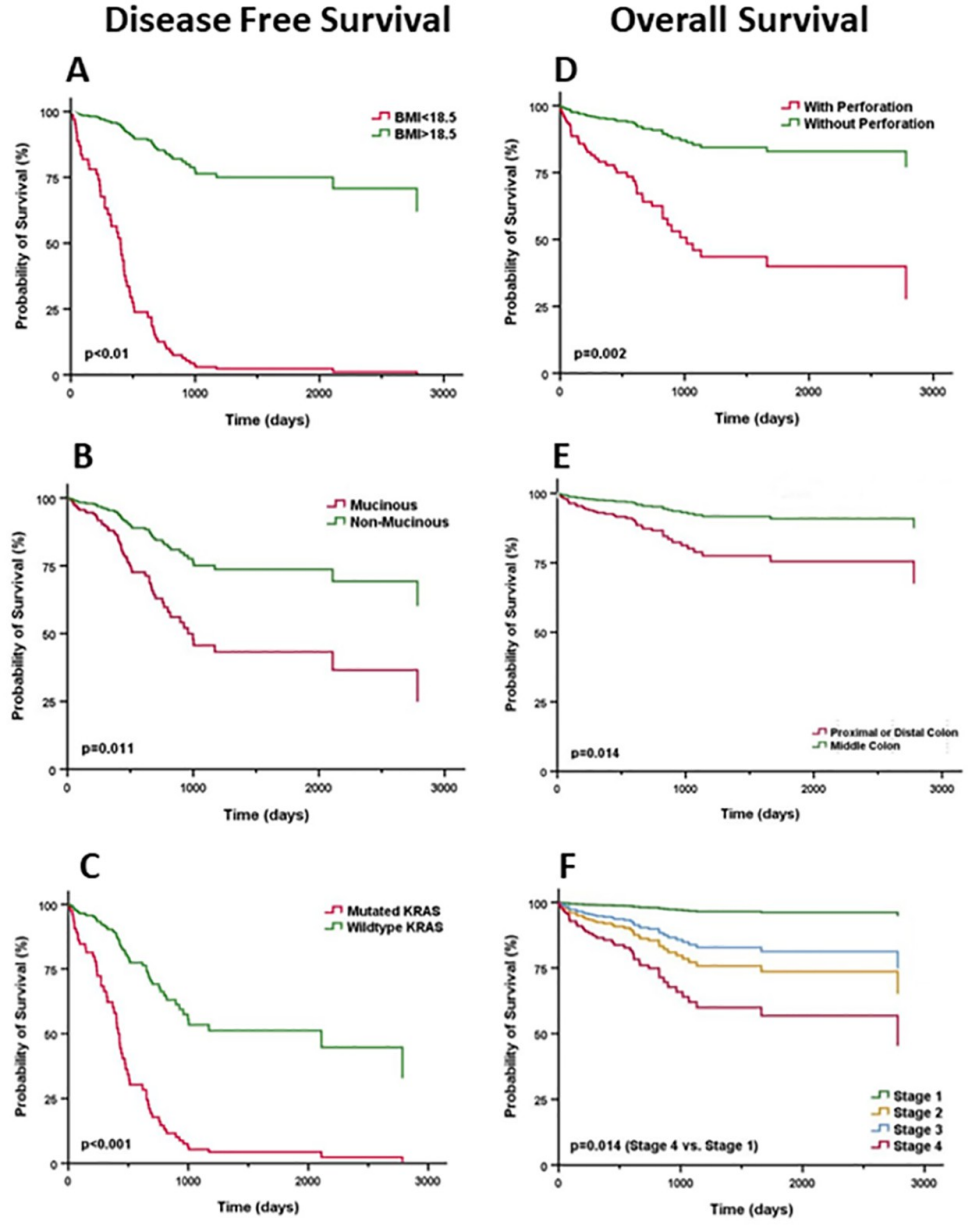
Impact of biological variables on survival of African American patients with CRC. A-C. Disease free survival relative to BMI (A), histologic type (B), and KRAS mutation (C). D-F. Overall survival relative to intestinal perforation (D), tumor location (E), and tumor stage (F).

To further determine the independent risk factors for prognosis of CRC in different racial groups, we introduced all trending variables with a p <0.2 in Kaplan-Meier analysis, into Multivariate Cox Proportional Hazard Model. After controlling for confounding factors, we found advanced stage, perforation and hypertension as significant variables adversely affecting the OS for both groups (AA and all-race). High CEA and LVI only predicted poor OS in all-race group. On the other hand, tumors from proximal or distal colon only significantly predicted poor OS in AA group (HR = 2.926, p = 0.014; Table 3; Fig 2).

**Table 3:** Multivariate cox regression analysis for overall survival (OS)*.

Variable	OS for All Races			OS for AA		
	HR	95% CI	p	HR	95% CI	p
**Stage (AJCC)**						
**1**	1			1		
**2**	3.949	0.793–19.656	0.093	7.903	0.925–67.506	0.059
**3**	2.461	0.502–12.079	0.267	5.359	0.647–44.396	0.12
**4**	5.96	1.176–30.196	0.031	14.583	1.722–123.512	0.014
**Intestinal Perforation**						
**Yes**	6.323	2.45–16.321	<0.001	4.901	1.789–13.432	0.002
**No**	1			1		
**Hypertension**						
**Yes**	2.634	1.35–5.142	0.005	2.173	1.076–4.388	0.03
**No**	1			1		
**Carcinoembryonic Antigen (ng/mL)**						
≥**5**	3.194	1.381–7.385	0.007			
**<5**	1					
**Lymphovascular Invasion**						
**Yes**	2.666	1.067–6.664	0.036			
**No**	1					
**Site**						
**Proximal or Distal**				2.926	1.24–6.904	0.014
**Middle**				1		

*Only values of statistically significant (p<0.05) variables are shown.

AJCC, American Joint Committee on Cancer; CI, confidence interval; HR, hazard ratio

In all-race group, AA had worse DFS than other races (HR = 3.682, p = 0.035). Low BMI, histology of non-mucinous adenocarcinoma (ADC), LVI, PNI, and KRAS mutation were all independent risk factors for poor DFS. No AA-specific factor for DFS was identified (Table 4).

**Table 4:** Multivariate cox regression analysis for disease free survival (DFS)*.

Variable	DFS for All Races			DFS for AA		
	HR	95% CI	p	HR	95% CI	p
**Race**						
**AA**	3.682	1.098–12.346	0.035			
**Non-AA**	1					
**Body-Mass Index (kg/m** ^**2**^ **)**						
**<18.5**	10.766	4.132–28.05	<0.001	13.066	4.636–36.823	<0.001
≥**18.5**	1			1		
**Histology**						
**Mucinous ADC**	2.451	1.146–5.244	0.021	2.743	1.259–5.977	0.011
**Non-Mucinous ADC**	1			1		
**Lymphovascular Invasion**						
**Yes**	2.549	1.385–4.691	0.003	2.554	1.344–4.853	0.004
**No**	1			1		
**Perineural Invasion**						
**Yes**	4.284	2.11–8.697	<0.001	4.847	2.278–10.315	<0.001
**No**	1			1		
**KRAS**						
**Wildtype**	4.178	1.787–9.772	0.001	4.669	1.979–11.015	<0.001
**Mutated**	1			1		

*Only values of statistically significant (p<0.05) variables are shown.

ADC, adenocarcinoma; CI, confidence interval; HR, hazard ratio

## Discussion

Racial disparities in survival of patients diagnosed with CRC have been extensively documented and the interplay of socioeconomic and biologic factors impacting the mortality from CRC is well known [4–9]. A multiple-match approach to determine the likely contribution of different factors to poor prognosis of AA patients has been investigated [15]. It is estimated that socioeconomic factors account for about 50% and tumor characteristics account for about 25% of racial disparity in mortality. It is noteworthy that those population-based studies focus more on social and behavioral causes of disparity than biological or pathological aspects. On the other hand, these studies did not explain the persisting racial disparity in mortality after adjusting for screening, healthcare and insurance, highlighting the need to elucidate the underlying biological basis [4–9, 15]. In the present study, we examined a wide range of demographic, clinical, pathological and molecular variables in CRC patients with different racial backgrounds, focusing on biological and pathological variables, to complement most previous population-based correlation studies. As expected, the DFS of AA patients was worse than the white counterparts, consistent with previous studies.

**Fig 3:**
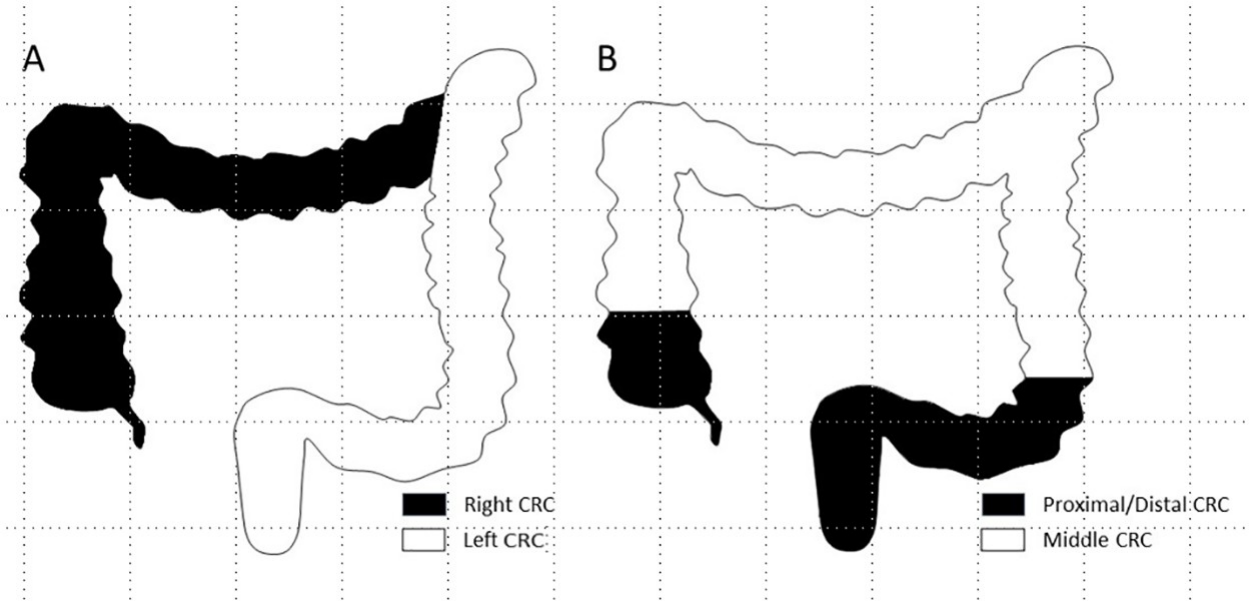
Illustration of anatomic distribution zones for CRC. A. Conventional laterality-based anatomic distribution (left and right) without significant association with prognosis; B. A newly proposed anatomic distribution (proximal/distal and middle) with significant correlation with prognosis in African Americans.

Conventionally, the CRCs arising in the right colon were found to be more prevalent in AA patients. A combination of hard-to-detect right-sided CRC and suboptimal access by colonoscopy may explain the worse prognosis in AA [16, 17]. However, our data did not identify poor prognosis for right-sided CRC as was identified by an earlier examination of the SEER data [18]. To determine if there is indeed an anatomic determinant with prognostic relevance, other than laterality, we redefined the anatomic distribution of CRC. A strong dependence of OS on tumor sub-sites defined as proximal (cecum)/distal (rectum and sigmoid colon) and middle (ascending colon, hepatic flexure, transverse colon, splenic flexure, and descending) colonwas observed in AA patients. Along the colorectal tract, the patients with CRC arising in either end have worse OS than those with CRC arising in mid colon (HR = 2.926, p = 0.014; Fig 3). Of interest, we did not note any difference between AA and white patient groups relative to re-defined anatomic localization. Although AA patients have a higher incidence of right CRC, as shown here and elsewhere [16, 17], this laterality difference is of no prognostic relevance, as mentioned earlier. Therefore, neither conventional locations and/or laterality nor the proposed anatomic locations play a role in racial disparity of CRC prognosis. The implication of this finding lies in that some pathogenic mechanisms underlying our re-defined anatomic locations may differ between AA and non-AA races, which is associated with distinct disease progression and hence prognosis.

Pathogenesis of CRC involves interactions between genetic and environmental factors and may also influence tumor localization as CRC in the same location have been shown to share molecular features. CRC with high microsatellite instability/CpG island methylator phenotype (MSI/CIMP) with BRAF/KRAS mutations are commonly seen on the right side, while CRC with low or negative chromosomal instability (CIN/CIMP) without KRAS mutations occur frequently on the left side [19]. Transcriptomic classifications, such as Consensus Molecular Subtypes (CMS), show enrichment of certain subtypes in different anatomic locations [20]. Environmental exposure (e.g., dietary factors and microbiota) also varies across different anatomic locations. For example, distal colon is more prone to genotoxic effect of fecal metabolites than the proximal colon. A recent study investigating risk factors associated with CRC at different anatomic locations found that each subsite, instead of simplified left/right colon, defines a distinctive risk factor profile [21]. Diets that activate inflammatory pathways are closely associated with CRC in both proximal and distal colon, while diets inducing hyperinsulinemia are more likely to cause CRC in the middle colon [21]. Right colon CRC has been reported to have a higher local failure rate after ablative radiotherapy suggesting distinct sensitivity of CRC at different anatomic sites to different treatment modalities [22]. Consistent with these observations, our findings support the prognostic value of the new system of anatomic delineation of CRC in AA patients.

Our findings also reveal other independent factors associated with poor survival. Most of these factors are also identified by other researchers as important predictors of CRC prognosis [23–27]. For example, low BMI represents a marker for decreased biological reserve and thus impaired capacity to compensate for the physical demands imposed by malignancy [23]. The serum level of CEA is positively correlated with tumor burden [24]. Presence of perforation, LVI or PNI indicates aggressive tumors [25–27]. After controlling for possible confounding variables, some factors which are significant in Kaplan-Meier survival analysis proved not to be independent predictors. It is noteworthy that although age does not have a significant effect on prognosis in multivariate analysis, we identified an unexpected turning point at the age of 40 years in Kaplan-Meier analysis. Forty to 49-year-old patients with CRC seems to have better prognosis among all age groups, whereas patients <40 years-old and those ≥50 years have shorter OS. These findings challenge the previously held viewpoint that patients <50-years have better prognosis than the older patients [28]. Previous studies suggested that CRC tends to occur at a younger age in AA than white patients [29]. Our findings support this conclusion when using 60 years as cutoff for CRC onset. Distinct pathogenic mechanisms may be at play in the white and AA patient populations, which warrant further investigation.

Several investigations have attempted to unravel the molecular mechanisms for racial differences in mortality from the perspective of gene expression profiling, microRNA profiling, or methylation patterns. A 15-gene mutation panel has been found to be associated with CRC in AA patients [30]. A different approach used by others focused on known carcinogenic abnormalities of CRC such as APC mutation, KRAS mutation, and MMR status [31–33]. It has been shown that there was no difference in frequency of microsatellite instability (MSI) between AA and white patients, although MSI is associated with a favorable prognosis. Also, KRAS mutation was reported to occur more frequently in AA patients with CRC. However, the prognostic relevance of KRAS mutation remains controversial [32, 33]. In addition, the previous genetic studies on KRAS and MMR status were limited by the small sample size of AA patients, which influenced the reproducibility of the results. In the context of these efforts, our study not only validated the distribution of KRAS and MMR status between AA and white patient groups, but also clarified the association of these molecular markers with prognosis of CRC in a larger AA patient population. We found that KRAS mutation frequency was higher in AA than in white patients, specifically, both in left colon CRC and proximal/distal colon CRC. Our study also identified KRAS mutation as an independent prognostic factor for inferior DFS. This difference in the frequency of KRAS mutation may form the biological basis for poor prognosis of CRC in AA patients. It is noteworthy that although in the CMS classification of CRC, KRAS mutation is enriched in the CMS3-metabolic subtype, our data do not suggest AA patients have more CMS3 subtypes of CRC, as KRAS mutation are also present in other subtypes in low frequency [20]. Therefore, transcriptomic analysis is needed to accurately classify and compare the CRC, and to better understand the racial disparity in prognosis.

Our study has some limitations. First, as an institution with an AA predominant patient population, our study required more matched white patients residing in the same area as controls. Although we used data of white patients on the East Coast and in similar year range from SEER database, variation in patient management exists in different institutions. Second, since this is a retrospective study, the data we collected may have selection bias, which means for some variables, their availability is probably limited to a certain group of patient population based on clinical judgement. Many variables of white patients were unavailable in SEER data and thus could not be included in our multivariate analysis. Therefore, more strictly controlled, preferably prospective, studies will address these limitations in the future.

In conclusion, we have proposed a new anatomic distribution which is better in predicting prognosis of CRC, particularly in AA patients, than previous laterality-based classification. As KRAS mutation is more frequently present in proximal/distal CRC in AA patients, it contributes to worse prognosis in these patients.

## Supporting information

S1 Table Kaplan-Meier analysis of variables associated with overall survival (OS; days, mean ± 1SE). (PDF)

S2 Table Kaplan-Meier analysis of variables associated with disease free survival (DFS; days, mean ± 1SE). (PDF)

S3 Table Frequency* of KRAS mutation among African American (AA) and white patients relative to conventional and newly proposed localization of colorectal cancer. (PDF)
