# Supplementary material for: Anatomic location of colorectal cancer presents a new paradigm for its prognosis in African American patients
Source: PLoS One. 2022 Jul 29;17(7):e0271629. doi: 10.1371/journal.pone.0271629 (PMC9337663; doi:10.1371/journal.pone.0271629)
Supplement: S1 Table — (PDF) [file pone.0271629.s001.pdf]

Table S1. Kaplan-Meier Analysis of Variables Associated with Overall Survival (OS; days, mean ± 1SE)

Variable	All-Race				African American			
	n	Days	% Event	p	*n*	Days	% Event	p
Gender								
Male	216	2390 ± 112	14.4		158	2276 ± 134	17.7	
Female	213	3739 ± 112	9.9	0.082	168	2688 ± 137	10.7	0.042
Age (Years)								
<40	13	1588 ± 133	7.7		11	1561 ± 158	9.1	
40-49	37	2888 ± 125	2.7		27	2834 ± 175	3.7	
50-59	112	2690 ± 151	9.8		75	2461 ± 164	13.3	
60-69	112	2615 ± 155	13.4		92	2592 ± 168	14.1	
≥70	155	2392 ± 152	15.5	0.037	121	2216 ± 158	17.4	0.133
Race								
AA	326	2577 ± 96	14.1					
White	42	2956 ± 158	4.8	0.375				
Body-Mass Index (kg/m^2)								
<18.5	22	1743 ± 335	22.7		17	944 ± 272	29.4	
≥18.5	347	2703 ± 87	11.2	0.025	268	2662 ± 99	12.7	0.002
Hypertension								
Yes	261	2574 ± 108	14.2		209	2547 ± 120	15.3	
No	168	2726 ± 126	8.9	0.178	117	2459 ± 152	12.0	0.473
Diabetes								
Yes	124	2725 ± 148	10.5		100	2753 ± 150	11.0	
No	305	2569 ± 99	12.8	0.202	226	2348 ± 110	15.5	0.107
Carcinoembryonic Antigen (ng/mL)								
<5	138	3001 ± 89	6.5		101	2908 ± 117	8.9	
≥5	139	1908 ± 172	19.4	<0.001	113	1955 ± 176	20.4	0.001
Anemia								
Yes	316	2584 ± 96	13.9		250	2516 ± 111	15.6	
No	92	2849 ± 143	7.6	0.096	65	2653 ± 141	9.2	0.141
Rectal Bleeding								
Yes	121	2264 ± 146	14.9		91	2171 ± 167	18.7	
No	308	2705 ± 93	11.0	0.446	235	2664 ± 106	12.3	0.22

Intestinal Obstruction								
Yes	60	2181 ± 215	15.0		50	2158 ± 247	14.0	
No	369	2670 ± 87	11.7	0.25	276	2599 ± 101	14.1	0.608
Intestinal Perforation								
Yes	18	1284 ± 229	38.9		14	1193 ± 253	42.9	
No	411	2693 ± 83	10.9	0.003	312	2637 ± 96	12.8	0.006
Site of Carcinoma								
Left colon	233	2692 ± 100	11.6		167	2453 ± 106	14.4	
Right colon	185	2599 ± 133	11.9	0.83	150	2574 ± 144	12.7	0.794
Cecum	76	2222 ± 165	14.5		61	2103 ± 193	18.0	
Ascending colon	71	2805 ± 193	7.0		61	2832 ± 203	6.6	
Transverse colon	26	2801 ± 184	7.7		21	2896 ± 171	4.8	
Descending colon	31	3118 ± 119	3.2		21		0	
Sigmoid colon	110	2610 ± 154	11.8		81	2140 ± 145	14.8	
Rectum	83	2423 ± 156	14.5	0.368	59	2342 ± 179	18.6	0.072
Proximal/Distal	269	2497 ± 108	13.4		201	2287 ± 111	16.9	
Middle	145	2885 ± 113	7.6	0.064	112	2936 ± 123	6.2	0.006
Histology								
Mucinous ADC	29	1799 ± 148	10.3		24	1756 ± 168	12.5	
Non-mucinous ADC	397	2641 ± 86	12.1	0.674	299	2581 ± 99	14.0	0.774
Grade								
WD	49	2739 ± 210	10.2		33	2454 ± 256	12.1	
MD	264	2700 ± 98	11.4		204	2634 ± 110	13.7	
PD/UD	44	1824 ± 217	20.5	0.119	34	1803 ± 230	23.5	0.106
Stage (AJCC)								
1	67	3081 ± 74	3.0		46	2962 ± 73	2.2	
2	69	2673 ± 142	10.1		52	2622 ± 171	11.5	
3	80	2803 ± 155	10		70	2769 ± 165	11.4	
4	83	1054 ± 175	22.9	<0.001	63	707 ± 66	25.4	<0.001
MMR Status								
MSI*	13				9			
MSS*	55				42			
KRAS								

Wildtype	43	1681 ± 287	20.9		36	1614 ± 315	22.2	
Mutated	52	1664 ± 192	26.9	0753	45	1602 ± 209	28.9	0.717
Lymphocytic Response								
Yes	85	2454 ± 107	7.1		69	2398 ± 127	8.7	
No	49	1963 ± 134	14.3	0.248	40	1944 ± 150	15.0	0.414
Lymphovascular Invasion								
Yes	63	2288 ± 217	14.3		50	2182 ± 244	18.0	
No	217	2883 ± 77	6.9	0.021	171	2762 ± 83	7.6	0.01
Perineural Invasion								
Yes	30	1487 ± 190	16.7		22	1212 ± 197	22.7	
No	237	2884 ± 74	6.8	0.023	188	2748 ± 80	8.0	0.07
Intratumoral Lymphocytic Infiltration								
Yes	70	2449 ± 120	7.1		57	2387 ± 144	8.8	
No	62	1984 ± 119	12.9	0.373	52	2157 ± 144	13.5	0.655
Peritumoral Lymphocytic Infiltration								
Yes	71	2277 ± 116	8.5		60	2210 ± 140	10.0	
No	60	2027 ± 116	11.7	0.658	49	2334 ± 148	12.2	0.973

*OS cannot be calculated because all MSI and MSS data are censored.

AA, African American; ADC, adenocarcinoma; AJCC, American Joint Committee on Cancer; MD, moderately differentiated, PD, poorly differentiated; SEER, Surveillance, Epidemiology and End Results program; UD, undifferentiated; WD well differentiated
