# Supplementary material for: Anatomic location of colorectal cancer presents a new paradigm for its prognosis in African American patients
Source: PLoS One. 2022 Jul 29;17(7):e0271629. doi: 10.1371/journal.pone.0271629 (PMC9337663; doi:10.1371/journal.pone.0271629)
Supplement: S2 Table — (PDF) [file pone.0271629.s002.pdf]

**Table S2.** Kaplan-Meier Analysis of Variables Associated with Disease Free Survival (DFS; days, mean ± 1SE)

| Variable | All-Race | | | | African American | | | |
|---|---|---|---|---|---|---|---|---|
| | *n* | Days | % Event | p | *n* | Days | % Event | p |
| Gender | | | | | | | | |
| Male | 158 | 2253 ± 133 | 18.4 | | 115 | 2146 ± 151 | 23.5 | |
| Female | 153 | 2275 ± 143 | 21.6 | 0.826 | 125 | 2151 ± 162 | 24.8 | 0.885 |
| Age | | | | | | | | |
| <40 | 10 | 1238 ± 177 | 30.0 | | 8 | 1206 ± 192 | 37.5 | |
| 40-49 | 27 | 1867 ± 270 | 25.9 | | 19 | 1675 ± 317 | 31.6 | |
| 50-59 | 86 | 2236 ± 192 | 19.8 | | 65 | 2010 ± 201 | 26.2 | |
| 60-69 | 87 | 2534 ± 172 | 16.1 | | 70 | 2516 ± 188 | 17.1 | |
| ≥70 | 101 | 2148 ± 173 | 20.8 | 0.72 | 78 | 1999 ± 186 | 25.6 | 0.532 |
| Race | | | | | | | | |
| AA | 240 | 2217 ± 114 | 24.2 | | | | | |
| White | 34 | 2381 ± 358 | 8.8 | 0.478 | | | | |
| Body-Mass Index (kg/m$^2$) | | | | | | | | |
| <18.5 | 15 | 1233 ± 370 | 40.0 | | 10 | 715 ± 253 | 50.0 | |
| ≥18.5 | 258 | 2428 ± 108 | 17.8 | 0.005 | 203 | 2332 ± 120 | 21.7 | <0.001 |
| Hypertension | | | | | | | | |
| Yes | 192 | 2358 ± 127 | 19.8 | | 156 | 2274 ± 139 | 23.1 | |
| No | 119 | 2207 ± 163 | 20.2 | 0.889 | 84 | 1997 ± 183 | 26.2 | 0.593 |
| Diabetes | | | | | | | | |
| Yes | 96 | 2386 ± 172 | 19.8 | | 79 | 2350 ± 181 | 22.8 | |
| No | 215 | 2244 ± 120 | 20.0 | 0.6 | 161 | 2038 ± 134 | 24.8 | 0.397 |
| Carcinoembryonic Antigen (ng/mL) | | | | | | | | |
| <5 | 117 | 2620 ± 132 | 16.2 | | 88 | 2483 ± 160 | 20.5 | |
| ≥5 | 82 | 1687 ± 203 | 26.8 | 0.004 | 68 | 1681 ± 207 | 30.9 | 0.028 |
| Anemia | | | | | | | | |
| Yes | 227 | 2250 ± 118 | 22.9 | | 178 | 2118 ± 133 | 27.5 | 0.066 |
| No | 77 | 2555 ± 186 | 11.7 | 0.085 | 62 | 2334 ± 166 | 14.5 | |
| Rectal Bleeding | | | | | | | | |
| Yes | 84 | 1880 ± 184 | 25.0 | 0.151 | 63 | 1728 ± 197 | 33.3 | 0.078 |
| No | 227 | 2421 ± 114 | 18.1 | | 177 | 2343 ± 129 | 20.9 | |
| Intestinal Obstruction | | | | | | | | |

| | | | | | | | | |
|---|---|---|---|---|---|---|---|---|
| Yes | 43 | 1460 ± 246 | 32.6 | | 37 | 1357 ± 266 | 32.4 | 0.06 |
| No | 268 | 2434 ± 105 | 17.9 | 0.017 | 203 | 2322 ± 118 | 22.7 | |
| Intestinal Perforation | | | | | | | | |
| Yes | 17 | 1309 ± 251 | 35.3 | | 13 | 1240 ± 283 | 38.5 | 0.17 |
| No | 294 | 2347 ± 105 | 19.0 | 0.119 | 227 | 2239 ± 117 | 23.3 | |
| Site of Carcinoma | | | | | | | | |
| Left colon | 157 | 2331 ± 135 | 18.5 | | 114 | 2156 ± 144 | 21.9 | |
| Right colon | 149 | 2251 ± 149 | 21.5 | 0.411 | 118 | 2146 ± 163 | 25.4 | 0.409 |
| Cecum | 64 | 2065 ± 184 | 20.3 | | 49 | 1895 ± 216 | 26.5 | |
| Ascending colon | 55 | 2386 ± 235 | 18.2 | | 49 | 2317 ± 249 | 20.4 | |
| Transverse colon | 21 | 2034 ± 343 | 28.6 | | 16 | 1972 ± 386 | 31.2 | |
| Descending colon | 27 | 2508 ± 289 | 14.8 | | 20 | 2315 ± 279 | 15.0 | |
| Sigmoid colon | 74 | 1799 ± 243 | 24.3 | | 53 | 1527 ± 193 | 30.2 | |
| Rectum | 48 | 2512 ± 185 | 12.5 | 0.274 | 38 | 2437 ± 210 | 15.8 | 0.293 |
| Proximal/Distal | 186 | 2165 ± 125 | 19.9 | | 140 | 2017 ± 135 | 25.0 | |
| Middle | 116 | 2405 ± 157 | 19.0 | 0.595 | 92 | 2326 ± 177 | 21.7 | 0.46 |
| Histology | | | | | | | | |
| Mucinous ADC | 29 | 1306 ± 188 | 31.0 | | 24 | 1230 ± 197 | 37.5 | |
| Non-mucinous ADC | 281 | 2388 ± 105 | 18.5 | 0.092 | 215 | 2290 ± 118 | 22.3 | 0.068 |
| Grade | | | | | | | | |
| WD | 33 | 2922 ± 178 | 6.1 | | 21 | 2622 ± 191 | 4.8 | |
| MD | 198 | 2328 ± 125 | 20.2 | | 158 | 2251 ± 137 | 24.1 | |
| PD/UD | 34 | 1492 ± 175 | 20.6 | 0.137 | 25 | 1105 ± 149 | 28.0 | 0.1 |
| Stage (AJCC) | | | | | | | | |
| 1 | 65 | 2703 ± 157 | 7.7 | | 44 | 2623 ± 195 | 9.1 | |
| 2 | 68 | 2149 ± 187 | 25.0 | | 51 | 2036 ± 217 | 29.4 | |
| 3 | 80 | 2032 ± 196 | 28.7 | | 70 | 1958 ± 203 | 32.9 | |
| 4 | 40 | 752 ± 69 | 22.5 | 0.027 | 30 | 746 ± 79 | 26.7 | 0.052 |
| MMR Status | | | | | | | | |
| MSI* | 13 | | 0 | | 9 | | 0 | |
| MSS* | 42 | | 16.7 | 0.085 | 33 | | 21.2 | 0.052 |
| KRAS | | | | | | | | |
| Wildtype | 31 | 1522 ± 313 | 29.0 | | 29 | 1377 ± 333 | 31.0 | |
| Mutated | 31 | 653 ± 95 | 64.5 | 0.011 | 24 | 600 ± 97 | 75.0 | 0.008 |
| Lymphocytic Response | | | | | | | | |

| | | | | | | | | |
|---|---|---|---|---|---|---|---|---|
| Yes | 83 | 1975 ± 154 | 19.3 | | 68 | 1906 ± 171 | 22.1 | |
| No | 49 | 1724 ± 150 | 22.4 | 0.829 | 40 | 1745 ± 160 | 22.5 | 0.921 |
| Lymphovascular Invasion | | | | | | | | |
| Yes | 63 | 1562 ± 228 | 31.7 | | 50 | 1474 ± 245 | 38.0 | |
| No | 214 | 2318 ± 110 | 17.3 | 0.003 | 169 | 2250 ± 122 | 20.1 | 0.004 |
| Perineural Invasion | | | | | | | | |
| Yes | 30 | 767 ± 150 | 43.3 | | 22 | 646 ± 151 | 54.5 | |
| No | 235 | 2304 ± 107 | 17.0 | <0.001 | 187 | 224 ± 119 | 20.3 | <0.001 |
| Intratumoral Lymphocytic Infiltration | | | | | | | | |
| Yes | 70 | 1962 ± 168 | 20.0 | | 57 | 1877 ± 191 | 22.8 | |
| No | 62 | 1732 ± 136 | 21.0 | 0.909 | 51 | 1749 ± 144 | 21.6 | 0.616 |
| Peritumoral Lymphocytic Infiltration | | | | | | | | |
| Yes | 69 | 1685 ± 137 | 18.8 | | 59 | 1483 ± 140 | 22.0 | |
| No | 60 | 1728 ± 137 | 21.7 | 0.892 | 46 | 1778 ± 144 | 21.7 | 0.647 |

*OS cannot be calculated because all MSI and MSS data are censored.

AA, African American; ADC, adenocarcinoma; AJCC, American Joint Committee on Cancer; MD, moderately differentiated; PD, poorly differentiated; SEER, Surveillance, Epidemiology and End Results program; UD, undifferentiated; WD, well differentiated
