# Supplementary material for: Anatomic location of colorectal cancer presents a new paradigm for its prognosis in African American patients
Source: PLoS One. 2022 Jul 29;17(7):e0271629. doi: 10.1371/journal.pone.0271629 (PMC9337663; doi:10.1371/journal.pone.0271629)
Supplement: S3 Table — (PDF) [file pone.0271629.s003.pdf]

Table S3. Frequency* of KRAS mutation among African American (AA) and white patients relative to conventional and newly proposed localization of colorectal cancer

	Conventional Anatomic Location				New Anatomic Location			
	Left		Right		Middle		Proximal/Distal	
	AA	White	AA	White	AA	White	AA	White
KRAS-Mu	28 (51.9)	169 (10.7)	20 (57.1)	825 (68.2)	15 (50.0)	507 (53.4)	33 (55.9)	487 (26.4)
KRAS-WT	26 (48.1)	1416 (89.3)	15 (42.9)	384 (31.8)	15 (50.0)	443 (46.6)	26 (44.1)	1357 (73.6)
P		<0.001		0.166		0.716		<0.001

*Expressed as n (%).
